# Efficacy and Security of Tetrodotoxin in the Treatment of Cancer-Related Pain: Systematic Review and Meta-Analysis

**DOI:** 10.3390/md21050316

**Published:** 2023-05-21

**Authors:** Miguel Á. Huerta, Javier de la Nava, Antonia Artacho-Cordón, Francisco R. Nieto

**Affiliations:** 1 Department of Pharmacology, University of Granada, 18016 Granada, Spain; huerta@ugr.es (M.Á.H.); javidelanava@gmail.com (J.d.l.N.); tartacho@ugr.es (A.A.-C.); 2 Institute of Neuroscience, Biomedical Research Center, University of Granada, 18016 Granada, Spain; 3 Biosanitary Research Institute ibs. Granada, 18012 Granada, Spain

**Keywords:** tetrodotoxin, cancer-related pain, chemotherapy-induced peripheral neuropathy, neuropathic pain, systematic review, meta-analysis

## Abstract

The pharmacological treatment of cancer-related pain is unsatisfactory. Tetrodotoxin (TTX) has shown analgesia in preclinical models and clinical trials, but its clinical efficacy and safety have not been quantified. For this reason, our aim was to perform a systematic review and meta-analysis of the clinical evidence that was available. A systematic literature search was conducted in four electronic databases (Medline, Web of Science, Scopus, and ClinicalTrials.gov) up to 1 March 2023 in order to identify published clinical studies evaluating the efficacy and security of TTX in patients with cancer-related pain, including chemotherapy-induced neuropathic pain. Five articles were selected, three of which were randomized controlled trials (RCTs). The number of responders to the primary outcome (≥30% improvement in the mean pain intensity) and those suffering adverse events in the intervention and placebo groups were used to calculate effect sizes using the log odds ratio. The meta-analysis showed that TTX significantly increased the number of responders (mean = 0.68; 95% CI: 0.19–1.16, *p* = 0.0065) and the number of patients suffering non-severe adverse events (mean = 1.13; 95% CI: 0.31–1.95, *p* = 0.0068). However, TTX did not increase the risk of suffering serious adverse events (mean = 0.75; 95% CI: −0.43–1.93, *p* = 0.2154). In conclusion, TTX showed robust analgesic efficacy but also increased the risk of suffering non-severe adverse events. These results should be confirmed in further clinical trials with higher numbers of patients.

## 1. Introduction

Pain is a very common symptom in cancer patients, with a prevalence of up to 66% [1]. Furthermore, it is very relevant since pain is one of the biggest concerns of cancer patients [2], as it is particularly disabling and affects the quality of life of these patients [3]. Cancer-related pain has a varied etiology and may be due to mechanisms related to the tumor growth itself (e.g., nerve compression or metastasis), chemotherapy treatment, or other therapeutic options (e.g., surgery or radiation) [4,5,6]. In fact, it is well known that pain associated with peripheral neuropathy is one of the main adverse effects of commonly used chemotherapeutic drugs such as paclitaxel, oxaliplatin, vincristine, and others [7]. This side effect, with a prevalence from 19% to 85% [8], potentially limits cancer treatment and consequently patient survival [9]. In many cases, the pain persists despite the end of chemotherapy and recovery from cancer [8,10].

The pathophysiology of cancer-related pain is very complex and difficult to classify [11]. It presents a nociceptive component associated with a neuropathic component that depends to a large extent on the characteristics of the tumor [12] or chemotherapy treatment [8,13]. The complexity of the pathophysiology of cancer-related pain also makes its treatment complex. The World Health Organization (WHO) and several clinical guidelines for the management of cancer pain recommend the use of paracetamol and nonsteroidal anti-inflammatory drugs (NSAIDs) for mild cancer pain, weak opioids for mild to moderate cancer pain, and strong opioids for the treatment of moderate to severe cancer pain [14,15,16]. Opioids can provide adequate analgesia, especially for higher cancer pain levels, but they produce severe adverse effects with large impacts on patients’ quality of life that are hard to manage, such as constipation [17]. First-line agents for neuropathic pain management (tricyclic antidepressants, serotonin–norepinephrine reuptake inhibitors, and gabapentinoids, among others) can also be used in cancer-related pain, mostly in neuropathic cancer pain [18,19,20]. Unfortunately, these drugs only possess moderate analgesic efficacy in neuropathic pain (30–50%) [21], and this is likely to be even lower for cancer-related pain [20]. They also produce important adverse effects [21]. A specific case of cancer-related pain is chemotherapy-induced peripheral neuropathy (CIPN), which is considered one of the most complex types of pain to treat, and currently there is no single pharmacological treatment with robust efficacy [8,22,23,24]. Given the lack of effective drugs for cancer-related pain, the development of new drugs that can improve its treatment with fewer adverse effects is necessary.

Tetrodotoxin (TTX) is a potent non-peptide guanidinium neurotoxin that specifically blocks voltage-gated sodium channels (VGSCs) [25]. TTX can be found in various marine and land animals, including pufferfish [26]. VGSCs are crucial in pain, and research has shown that some types of TTX-sensitive VGSCs are directly related to chronic pain, especially neuropathic pain [27,28]. Preclinical evidence has shown that the systemic administration of TTX has efficacy in classical models of pain [29], in a model of carrageenan-induced inflammatory mechanical hyperalgesia [30], and in different models of visceral pain [31]. In addition, TTX reduced pain behaviors in different models of neuropathic pain, including neuropathic pain induced by nerve injury [29,32], postherpetic neuralgia [33], and chemotherapy-induced neuropathic pain [34,35], as well as in a model of bone cancer pain [36]. On the other hand, this drug has completed, with some degree of efficacy, several clinical trials for the treatment of cancer pain (NCT00725114 and NCT00726011) and chemotherapy-induced neuropathic pain (NCT01655823) [24,37].

The main objective of this systematic review was to evaluate the available clinical evidence regarding the efficacy and safety of TTX for the treatment of cancer-related pain. Additionally, we aimed to quantify the size of the effect by conducting a meta-analysis.

## 2. Results

### 2.1. Study Selection

**Figure 1:**
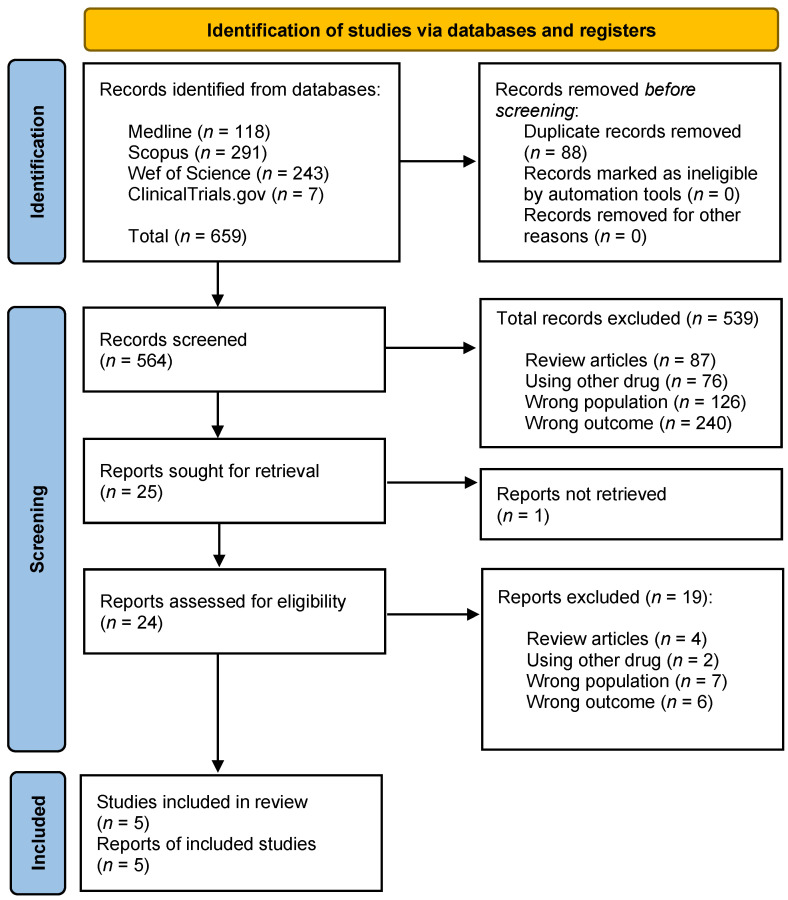
Study selection flow diagram.

A summary flow chart is shown in Figure 1. The search yielded 659 references. After removing duplicates, we obtained 564 articles. Then, the titles and abstracts were evaluated according to the “Inclusion and Exclusion Criteria” (see Section 5.4), and as a result, 539 articles were excluded: 87 review articles, 76 for not evaluating TTX (articles that used other drugs), 126 for assessing a different population (laboratory animals), and finally, 240 for a wrong outcome (articles mainly describing TTX intoxications). Of the 25 remaining full-text articles that were assessed for eligibility, 20 were excluded for different reasons: 1 article because the full text was not available, 2 articles because the drug used was not TTX, 7 studies because the population was not adequate, 6 articles for reasons related to the reported outcome, and 4 articles because they were reviews. Finally, we selected a total of 5 studies for this systematic review, which were the only clinical articles that evaluated TTX to treat any form of pathological pain in humans. They evaluated the efficacy of TTX to alleviate neuropathic pain induced by cancer chemotherapy or cancer-induced pain, which are both considered cancer-related pain [38,39,40,41,42].

### 2.2. Study Characteristics

Detailed characteristics of the included studies and information about efficacy are shown in Table 1. The studies were published from 2007 to 2021 in four countries: Canada, Australia, New Zealand, and the United States of America. Three of them were randomized clinical trials (RCTs) (Hagen et al. [31], Hagen et al. [33] (phase III; NCT00725114), and Goldlust et al. [34] (phase II; NCT01655823)), and the other two were non-controlled clinical trials (Hagen et al. [30] (phase IIa) and Hagen et al. [32], which was an open-label study conducted in patients who had previously participated in the RCT described in [31]). There was another clinical trial (NCT00726011), the results of which have not been published, that provided the option for all patients who participated in the TEC-006 study (NCT00725114) [41], both TTX- and placebo-treated, to receive or continue to receive TTX treatment. All clinical trials were conducted by Wex Pharmaceuticals Inc. for cancer pain [38,39,40,41] and chemotherapy-induced neuropathic pain [34]. Although intramuscular injection was tested in the first dose-escalation non-controlled trial published in 2007 [38], the most common administration route for TTX was subcutaneous injection, which was used in later clinical trials [39,40,41]. The most common dose was 30 μg, which was included in all completed trials and was the dose selected for the ongoing clinical trial (NCT05359133). Table 2 summarizes the main adverse effects detected in the different clinical trials carried out.

### 2.3. Meta-Analysis

#### 2.3.1. Efficacy of Tetrodotoxin in RCTs

**Figure 2:**
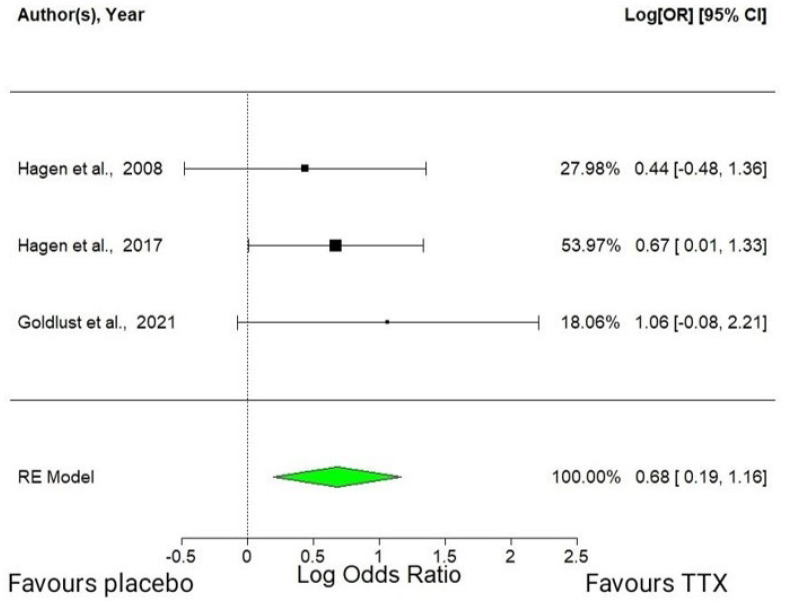
Forest plot of the analgesic efficacy of TTX in cancer-related pain [39,41,42]. Forest plot depicting the increase in patients achieving a ≥ 30% reduction in the mean pain intensity, as measured using the log odds ratio in a random-effects model. CI, confidence interval; RE, random-effects.

This meta-analysis used a random-effects model with the three available RCTs. Tetrodotoxin administered at a dose of 30 μg twice a day (BID) was found to have an average effect size of 0.68 (95% CI: 0.19–1.16, *p* = 0.0065). This statistically significant positive effect means that TTX increased the number of patients who achieved an improvement ≥30% in the mean pain intensity versus placebo (Figure 2). The estimated amount of total heterogeneity (tau²) was 0, indicating that there was no significant variation in the effect sizes across the studies beyond what would be expected by chance. The total heterogeneity/total variability (*I²*) was 0.00%, indicating low heterogeneity. However, caution should be used when interpreting these results because of the small number of studies included in the analysis.

#### 2.3.2. Security of Tetrodotoxin in RCTs

**Figure 3:**
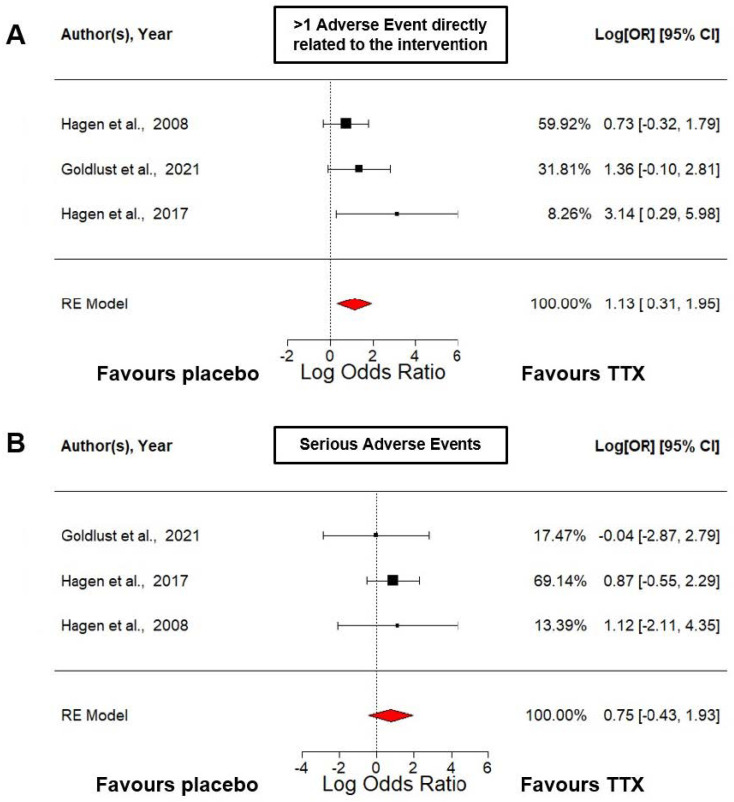
Safety of TTX in cancer-related pain RCTs analyzed using a random-effects model [39,41,42]. (**A**) This forest plot shows the log odds ratio versus placebo of suffering at least 1 adverse event related to the intervention. (**B**) This forest plot shows the log odds ratio versus placebo of suffering any serious adverse event. CI, confidence interval; RE, random-effects.

The first meta-analysis regarding security (Figure 3A) assessed the risk of suffering at least one non-severe adverse effect related to the intervention. It was performed using a random-effects model that estimated an overall effect size of 1.13 (95% CI: 0.31–1.95, *p* = 0.0068). This suggests that there was a positive association between the treatment with TTX administered at a dose of 30 μg BID and suffering at least one non-severe adverse effect related to the intervention. The standard error (SE) was 0.42, which means that there was some uncertainty around this estimate. There appeared to be no significant heterogeneity among the included studies (*I²* = 0.00%). However, it is important to note that the estimated amount of total heterogeneity (tau²) was quite small and the SE was quite large, which may limit the precision of this estimate.

The second meta-analysis regarding security (Figure 3B) assessed the risk of suffering a serious adverse effect during the treatment with TTX or placebo. It was performed using a random-effects model that estimated an overall effect size of 0.75 with a wide 95% confidence interval (−0.43 to 1.93), which was not statistically significant (*p* = 0.2154). Therefore, based on this analysis, we cannot conclude that TTX administered at a dose of 30 μg BID increased the risk of suffering a serious adverse effect.

However, caution should be used when interpreting these results because of the small number of studies included in the analysis.

### 2.4. Risk of Bias

**Figure 4:**
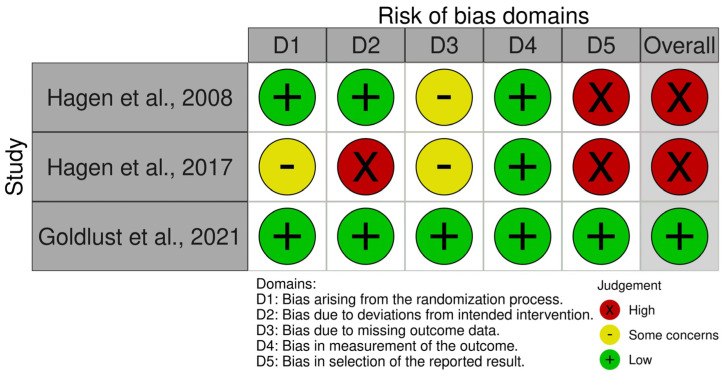
Summary of the risk of bias assessment of the RCTs with the Risk of Bias 2 (Rob2) tool [39,41,42].

A summary of the risk of bias analysis for the three RCTs included in the meta-analysis, as assessed using the Risk of Bias 2 (RoB2) tool, is shown in Figure 4.

Hagen et al. [39] showed some concerns in two domains (domain 1: bias arising from the randomization process and domain 3: bias due to missing outcome data), and high risk in domain 5 (due to bias in the selection of the reported result). In terms of randomization, the study provided insufficient details regarding the allocation sequence and the methods used for concealing treatment allocation. As for the reported results, the study did not provide details on the number of randomized participants or the number of participants who completed the study, and the national clinical trial (NCT) identifier number was missing. For domain 3, data for this outcome were not available for all randomized participants. In this domain, large losses are considered to underestimate the effects; although it is true that in this case the losses slightly exceeded 5% in the TTX group and were not reached in the placebo group (the availability of data was 92.6% for the TTX group and 95.1% for the placebo group). In the analysis of domain 5, we found that analgesic responses were assessed using descriptors 1–8 of the Brief Pain Inventory (BPI) scale; the 6 descriptors of the McGill Pain Questionnaire (MPQ); the 10 descriptors of the Neuropathic Pain Scale (NPS) (when applicable); the 0–10 visual analog scale; and a patient diary, which included the characterization and assessment of pain intensity (on a 0–10 scale) of the three most bothersome pains, a review of concurrent medication, and the patient’s impression of change, so it is clear that they reported multiple eligible outcome measurements within the outcome domain. Therefore, we identified a high risk of bias in this study.

Hagen et al. [41] had some concerns in the same two domains as Hagen et al. [39] and a high risk of bias in domains 2 and 5 (bias due to deviations from the intended intervention and bias in the selection of the reported result). Although the study reported adequate methods for randomization and provided an NCT identifier number, the allocation concealment was unclear. In relation to bias due to missing outcome data, the availability of data was 84.4% for the TTX group and 95.5% for the placebo group, so in this case they greatly exceed the limit of 5% in the TTX group. In relation to domain 2, while it is to be expected that deviations may have occurred outside the context of the trial, it should be mentioned that although the study claimed that participants were blinded to treatment assignment, there was a high rate of symptoms of drug-specific adverse effects in the TTX group and that the number of withdrawals due to adverse events was higher in the TTX group than in the placebo group. This could have made the participants aware of the treatment group. It should be added that while the intent was to enroll 254 patients, because of associated costs, the sponsor decided to halt the study’s enrollment at 165 patients. Along the same lines as the previous study, they also reported multiple eligible outcome measurements within the outcome domain. Therefore, we also identified a high risk of bias in this study.

On the contrary, Goldlust et al. [42] did not show any concerns in any of the domains, including domain 3 (bias due to missing outcome data), as the availability of data was 96% for the TTX at 30 μg twice a day (BID) group and 100% for the placebo group. Therefore, the overall risk of bias judgment for this report is low.

It is important to note, however, that the absence of bias does not necessarily imply that the results are generalizable to other populations or contexts. Further research may be needed to validate the findings of these studies in other settings.

In addition, we used the Risk of Bias In Non-Randomized Studies of Interventions (ROBINS-I) tool to assess risk of bias in the two non-controlled studies that were selected. The results are summarized in Supplementary Data S1 (Table S1).

### 2.5. Publication Bias

**Figure 5:**
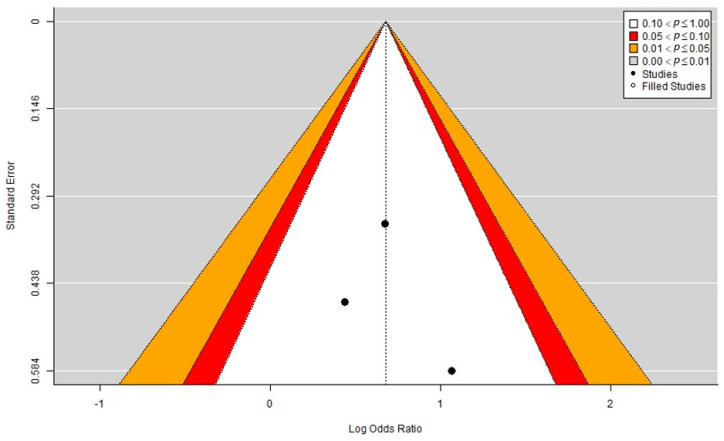
Funnel plot analyzing publication bias, including the trim and fill method.

In our analysis (Figure 5), the funnel plot showed a generally symmetrical distribution of studies around the estimated effect size, indicating a low risk of publication bias. Additionally, no points were outside the funnel limits or close to the boundary, suggesting that there were no significant outliers or influential studies. Furthermore, we used the trim and fill method to test whether there were any missing studies on the right side of the funnel plot, which could indicate the presence of publication bias. However, the method did not find any studies to fill the gap, indicating that the observed effect size was not significantly influenced by missing studies. Therefore, based on the funnel plot and the trim and fill analysis, we concluded that there is a low risk of publication bias for this meta-analysis and that the evidence base is likely to be reliable and robust.

## 3. Discussion

Pain is one of the main symptoms of cancer patients, and currently its treatment is not satisfactory. Hence, the development of new therapies (especially with non-opioid drugs) to alleviate this type of pain is a real need. The beneficial effects of TTX in chronic pain have been evaluated at the preclinical and clinical levels [27,28,37]. However, to the best of our knowledge, this systematic review and meta-analysis is the first to evaluate the effects of treatment with TTX in patients with chronic pain and, in particular, pain as a consequence of cancer or its treatment. Our overall results suggest that TTX treatment could reduce cancer-related pain without producing serious adverse events in patients.

In this systematic review, five clinical trials were collected to evaluate the effect of TTX on cancer-related pain, including pain that may be caused by the cancer itself or by chemotherapy. There are no other clinical articles that have evaluated the efficacy of TTX to treat other forms of pathological pain. The results of the meta-analysis include three RCTs showing that TTX at a dose of 30 μg twice a day (BID) significantly increased the number of patients who achieved an improvement ≥30% in the mean pain intensity versus placebo. The quantitative analyses included in this meta-analysis were performed using only data from patients who were treated with the dose of 30 μg BID or placebo, as this dose and schedule were used in all analyzed studies, which included a total of 298 patients. Moreover, the efficacy of TTX in the treatment of cancer-related pain was also supported by results from patients included in these RCTs with different dosages and regimens. For example, in Goldlust et al. 2021 [42], 25 patients were treated with 7.5 μg BID, 24 were treated with 15 μg BID, and 26 were treated with 30 μg BID. When we compared the efficacy of these groups versus placebo, all groups showed moderate dose-dependent increases in the number of responders to the primary outcome: 36.0%, 45.8%, and 57.7.0%, respectively, versus placebo (32.0%). In addition, in this parallel dose-finding RCT, the dose-dependent effect of TTX was also demonstrated, which can be summarized using the odds ratios versus placebo: 1.12 (7.5 μg BID), 1.99 (15 μg BID), and 3.39 (30 μg BID) [42]. Another important finding described in these RCTs, which supports the efficacy of this drug, is the long-acting analgesia following TTX administration in comparison to placebo [39,41], and the patients also showed improvements in other secondary outcomes related to quality of life or reductions in opioid consumption [39,41,42]. Moreover, there were other non-controlled clinical experiences with TTX that showed efficacy in cancer-related pain patients [38,40]. In one of these studies, after the completion of the RCT called TEC-006 (NCT00725114), a new open-label clinical trial (NCT00726011) was started in which the same patients were offered to start or continue treatment with TTX at 30 μg BID. In this study, 45 patients were enrolled, and the efficacy achieved by the TTX treatment (in terms of the number of responders) was 47%, with a relief of cancer pain that was persistent during successive treatment cycles without any evidence of tolerance [40].

The mechanism of action of TTX, the blockade of VGSCs, which play a key role in pain transmission, explains its effects in relieving pain [27]. However, TTX has good selectivity (at therapeutic doses) for some VGSC isoforms (e.g., Na_v_1.6 and Na_v_1.7) that are highly expressed in the nervous system, and in different studies such isoforms have been reported to be upregulated in chronic pain states (including neuropathic or cancer-related pain) [43,44,45,46]. In addition, there are noteworthy findings that demonstrate the massive re-expression of the Na_v_1.3 isoform in adult primary sensory neurons following nerve injury since this channel is not normally expressed in the peripheral nervous system (except during the embryonic phase) [47,48,49,50]. The selectivity of TTX for some VGSC isoforms and the changes in the expression of such TTX-sensitive VGSCs, which can generate abnormal activity in the nervous system during pathological pain, probably explain the efficacy of TTX in relieving cancer-related pain described here.

The analgesic efficacy of TTX has been evaluated at a preclinical level in several models of pathological pain, mainly neuropathic pain of different etiologies [29,32,33,34] but also bone cancer pain [36] and visceral pain [31], among others. In addition, as mentioned above, numerous studies, mainly at a preclinical level, have shown that TTX-sensitive VGSCs are highly involved in the pathophysiology of pathological pain [27]. Altogether, the preclinical studies and the promising results shown here at the clinical level for cancer-related pain suggest that TTX could also be a good therapeutic strategy for the treatment of other types of pathological pain in patients, such as neuropathic pain (not only that related to cancer). However, this has not been evaluated to date.

The evaluation of the safety of TTX is a key aspect since this compound is a potent neurotoxin that is responsible for many human intoxications, mainly after the ingestion of pufferfish [26]. However, all clinical experiences evaluated in this meta-analysis suggest that subcutaneous treatments with TTX do not increase the risk of suffering a serious adverse effect, but it has a positive association with suffering at least one non-severe adverse effect related to the intervention. It seems that almost all adverse effects associated with this drug can be considered mild or moderate, with sensory alterations and gastrointestinal issues being the most common. In addition, when we looked at the mortality during all the clinical trials included here, we found that no deaths related to TTX were reported, and two patients died in the placebo group in one of the studies [38], probably due to cancer.

Another important aspect to consider with a VGSC blocker such as TTX is the potential cardiac toxicity because VGSC isoform Na_v_1.5 is responsible for the upstroke of the action potential in the heart [51]. Interestingly, Na_v_1.5 is considered a TTX-resistant VGSC that is not blocked by this drug at therapeutic doses [27]. This is a great advantage of TTX over other non-selective VGSC blockers that can inhibit the cardiac Na_v_1.5 isoform at doses closer to the therapeutic ones, such as local anesthetics, which are known to cause cardiac toxicity. In fact, a recent phase I dose-escalation study evaluated the safety and tolerability of TTX at clinically relevant exposures in healthy volunteers, showing that TTX was well tolerated and did not produce any clinically significant impairments in the cardiac, neuromuscular, or respiratory systems [52].

The most important limitation of our results is the sample size. There were only three small RCTs that evaluated TTX for cancer-related pain involving a total of 372 patients. Of these, we only included patients treated with the dose of 30 μg BID (298 patients, which represented the majority of them). Because of this small number, we obtained a wide confidence interval for the heterogeneity analysis (*I²*: 0%; 95% CI: 0.0–94.7), which indicated some uncertainty in these estimates, although we interpreted them as low heterogeneity because we obtained a *p*-value = 0.7039. A positive point is that the sizes of the three RCTs included here were similar since it is known that unbalanced sizes of studies is a big limitation when conducting a meta-analysis with few studies [53].

The good efficacy and safety profile that TTX has been shown to have in the treatment of cancer-related pain is very promising but needs to be confirmed with new RCTs with higher numbers of patients. According to the WEX Pharmaceuticals Inc. website, a phase III RCT using TTX to treat chemotherapy-induced neuropathic pain is in the process of launching in the United States and Canada. We also found on clinicaltrials.gov that a new phase II RCT using TTX at a dose of 30 μg twice a day to treat chemotherapy-induced neuropathic pain involving 222 patients is already ongoing and is estimated to be completed by the beginning of 2025 (NCT05359133).

## 4. Conclusions

TTX showed robust analgesic efficacy but also increased the risk of suffering non-severe adverse events. However, TTX did not increase the risk of suffering a severe adverse event. These results should be confirmed in further clinical trials with higher numbers of patients. Therefore, we will have to wait some time to confirm whether TTX finally reaches patients with cancer-related pain, who have a huge need for a truly effective medicine to relieve their pain.

## 5. Materials and Methods

### 5.1. Protocol and Registration

The methodology used in this review was specified in advance and documented in a protocol that was registered on the CRD (Centre for Reviews and Dissemination) York website International Prospective Register of Systematic Reviews (PROSPERO) under the registration ID CRD42023403129. The review was performed adhering to the latest version (2020) of the Preferred Reporting Items for Systematic Reviews and Meta-Analyses (PRISMA) guidelines on systematic reviews and meta-analyses [54].

### 5.2. PICO Research Question

The PICO format is broadly used in evidence-based clinical practice:

(P) Adults with cancer-related pain.

(I) Treatment with tetrodotoxin, also known as TTX, tetradotoxin, fugu toxin, or tarichatoxin.

(C) Not controlled and placebo-controlled studies.

(O) Pain reduction, quality-of-life improvement, and side effects.

### 5.3. Information Sources and Search Strategy

We performed a comprehensive systematic search in four databases (Medline, Web of Science, Scopus, and ClinicalTrials.gov) without restrictions for the language or date. The search was updated on 2 May 2023. The search strategies for each database were as follows:

Medline: “Tetrodotoxin” [Mesh] OR “Tetrodotoxin” [tiab] OR “TTX” [tiab] OR “Tetradotoxin” [tiab] OR “Fugu Toxin” [tiab] OR “Tarichatoxin” [tiab]. Filters: case reports and clinical trials.

Web of Science: AB = ((tetrodotoxin OR TTX OR Tetradotoxin OR Fugu Toxin OR Fugu Toxin) AND (clinical trial OR case report* OR Clinical Study)).

Scopus: TITLE-ABS-KEY ((tetrodotoxin OR TTX OR tetradotoxin OR “fugu toxin”) AND (“clinical trial” OR “clinical study”)).

### 5.4. Inclusion and Exclusion Criteria

Inclusion criteria: original human clinical studies (including clinical trials) in which adult patients with chemotherapy-induced neuropathic pain or cancer pain were treated with tetrodotoxin, also known as TTX, tetradotoxin, fugu toxin, or tarichatoxin.

Exclusion criteria: review articles, systematic evaluations, in vitro experiments, animal studies, studies including no relevant information or missing information, some ongoing clinical trials with no published results, and violations of any of the above inclusion criteria.

### 5.5. Article Selection

Titles and abstracts of studies were retrieved using the search strategy by two review authors (MAH and JDLN) in a blind manner to identify studies that potentially met the inclusion criteria. The full texts of these potentially eligible studies were retrieved and independently assessed for eligibility by two review team members (MAH and JDLN). The selection process was carried out using the software Rayyan. Any disagreement between the authors over the eligibility of particular studies was resolved through a discussion with a third reviewer (FRN).

### 5.6. Data Extraction

The extracted information included the study setting; the study population and baseline characteristics; details of the intervention, including the strategy used, the timing, the dose used, and the control conditions; the study methodology; outcomes related to pain explaining the pain assessment method used, quality of life, and adverse events; the main results of the intervention; and information for the assessment of the risk of bias. Two review authors (MAH and JDLN) extracted data independently (blind), and discrepancies were identified and resolved through discussion (with a third author (FRN), where necessary).

### 5.7. Meta-Analysis and Statistics

The meta-analysis was performed using open-access software: R-4.1.3 and RStudio2022.07.2. The full code is available as text in Supplementary Data S2. The meta-analysis included in this manuscript combined the results of 3 randomized controlled trials reporting the efficacy and security of TTX administered at the dose of 30 μg twice a day and was conducted using a random-effects model. The random-effects model considers both within-study and between-study variability and assumes that the true effect size may vary from study to study. To calculate the sizes of the effects, we used the log odds ratio (logOR), a common measure used in meta-analyses of binary outcomes that quantifies the association between the exposure and the outcome. The logOR has some advantages over the OR: (1) it is a continuous variable, and as such, it is easier to standardize and compare between studies; (2) the logarithmic transformation helps to stabilize the variance of the estimate, which is useful when pooling studies with different sample sizes or effect measures; and (3) the logOR has a symmetric distribution that is closer to the normal distribution than the OR, which can be useful for statistical inference and hypothesis testing. To calculate the logOR, one needs to take the natural logarithm of the OR. The logOR formula is defined as follows: logOR = ln(OR) = ln(ad/bc), where a, b, c, and d represent the cell frequencies in a 2 × 2 contingency table. A heterogeneity assessment was performed to evaluate the degree of variation among the effect sizes of the individual studies. The presence of heterogeneity in a meta-analysis means that the effect sizes of studies vary more than one would expect due to random variation alone. We used the Q-test method, which calculated the observed heterogeneity between studies as the difference between the overall effect size and the effect sizes of the individual studies. The test assumed that the effect sizes followed a normal distribution, and the null hypothesis was that there was no statistical heterogeneity among the studies. If the obtained *p*-value was less than a predetermined significance level (e.g., 0.05), we rejected the null hypothesis and concluded that there was significant heterogeneity among the studies. The degree of heterogeneity was quantified using the statistical values *I^2^* and *H^2^*. Statistical significance was considered when *p* < 0.01.

### 5.8. Risk of Bias Assessment

Two reviewers (AAC and FRN) independently evaluated the risk of bias at the study level, and discrepancies were identified and resolved through discussion (with a third author (MAH), where necessary). The risk of bias was assessed using two different methods: (1) RoB2 (version 2 of the Cochrane risk-of-bias tool for randomized controlled trials). This tool is structured into five domains through which bias might be introduced into a result. An evaluation is assessed into one of 3 categories: high risk of bias, some concerns, and low risk of bias. An assessment is specific to a single trial result that is an estimate of the relative effects of two interventions or intervention strategies on a particular outcome. (2) ROBINS-I. The Risk Of Bias In Non-Randomized Studies of Interventions tool was used for non-randomized studies of interventions. The tool has seven domains for appraising non-randomized observational studies such as cohort studies, case-control studies in which intervention groups are allocated during the course of usual treatment decisions and subsequently lack detail, and quasi-randomized studies in which the method of allocation falls short of full randomization. The evaluation is expressed in terms of 5 outcomes from low risk of bias to critical risk of bias or no information on which to base a judgement. A RoB2 summary plot (Figure 4) was created using the code provided by [55].

### 5.9. Publication Bias

Potential publication bias was assessed through Egger’s and Begg’s tests [56]. The trim and fill method is a nonparametric (rank-based) data augmentation technique that was proposed by Duval and Tweedie [57]. This method can be used to estimate the number of studies missing from a meta-analysis due to the suppression of the most extreme results on one side of a funnel plot. The method then augments the observed data so that the funnel plot is more symmetric.

## Figures and Tables

**Table 1:** Summary of the efficacy of TTX in clinical trials for cancer-related pain.

Study/Clinical Phase/Condition	Number/Country	Administration/ Doses	Efficacy in Primary Outcome	Secondary Outcomes
Hagen et al., 2007 [38] Phase IIa SCP	24 Canada	i.m. injections/ 4 days of 7.5, 15, 22.5, and 30 μg BID or 30 μg TID	In total, 17 of 31 (54.85%) patients had a ≥33% reduction in the intensity of the worst, average, or current pain for at least two consecutive days.	For BPI#9A (general activity), 13 responders and 4 non-responders improved. For BPI#9G (enjoyment of life), 12 responders and 0 non-responders improved.
Hagen et al., 2008 [39] MSCP	82 Canada	s.c. injections/ 4 days of 30 μg BID or placebo	In total, 16 of 38 (42%) patients in the TTX arm and 12 of 39 (31%) patients in the placebo arm were responders.	Improvements in pain or significant reductions in opioid use, along with improvements in QoL, were observed in 17 of 38 (45%) responders to TTX and 8 of 39 (21%) responders to placebo. The duration of analgesia was 19.5 days in the intervention group versus 14.3 in the placebo group (NNT: 4.2).
Hagen et al., 2011 [40] MSCP	45 Canada	s.c. injections/ 4 days of 30 μg BID	There were 16 (39%) responders, 5 (12%) clinical responders, and 20 (49%) non-responders to TTX in treatment cycle 1. In subsequent cycles, the effect on pain did not change.	After the first cycle of TTX at 30 μg BID for 4 days, pain relief lasted, on average, 21 days. The average duration of pain relief remained at about 20 days for all subsequent cycles.
Hagen et al., 2017 [41] Phase III MSCP	165 Australia, Canada, and NZ	s.c. injections/ 4 days of 30 μg BID or placebo	In total, 33 (50.8%) patients who received TTX and 29 (34.5%) patients who received a placebo were responders.	When adding QoL to the analysis, the proportions of responders to treatment with TTX during EPIP or LPIP were not statistically significant. The duration of the analgesic response was 56.7 days in the intervention group versus 9.9 days in the placebo group (NNT: 6.2).
Goldlust et al., 2021 [42] Phase II CINP	125 USA	s.c. injections/ 4 days of 7.5, 15, and 30 μg BID, 30 μg QD, or placebo	Numbers of responders: 7.5 µg BID: 9 (36.0%), 15 µg BID: 11 (45.8%), 30 µg QD: 10 (40.0%), 30 µg BID: 15 (57.7%), and placebo: 8 (32.0%).	On day 28, there were benefits of TTX at 15 μg BID and 30 μg BID on all outcome subscales of the SF-36 and the sensory and motor nerve function subscales of the EORTC CIPN20.

SCP: severe cancer pain; MSCP: moderate to severe cancer pain; CINP: chemotherapy-induced neuropathic pain; NZ: New Zealand; TTX: tetrodotoxin; BID: twice daily; TID: three times a day; QD once a day; i.m.: intramuscular; s.c.: subcutaneous; NNT: number to treat; QoL: quality of life; BPI: Brief Pain Inventory; EORTC CIPN20: European Organization for Research and Treatment of Cancer Chemotherapy-Induced Peripheral Neuropathy 20; EPIP: Early post-injection period; LPIP: late post-injection period.

**Table 2:** Summary of adverse events (AEs) of TTX in clinical trials for cancer-related pain.

Study	Mild Adverse Events	Severe Adverse Events
Hagen et al., 2007 [38] Phase IIa SCP	All 24 subjects in the 31 treatments experienced AEs (531 reported). Approximately 98% of the total were rated as mild, with paresthesia and hypesthesia the most repeated, followed by nausea. These AEs appeared to increase in frequency in a dose-dependent manner.	AEs in the 30 µg TID dose group led to three patients discontinuing treatment partway through the four-day treatment period and a subsequent decision to discontinue further dose escalation. One of these AEs was an episode of severe ataxia after the seventh injection of TTX, which resolved within 24 h.
Hagen et al., 2008 [39] MSCP	In total, 648 of 690 AEs were considered mild or moderate, and 38 of 41 patients in the TTX group presented one or more AEs (92.7%). Overall, treatment-emergent AEs were greater in the TTX arm than in the placebo arm, but almost all were mild and related to tingling, numbness, or other transient sensory symptoms.	Three patients discontinued TTX (1) due to moderately severe but transient ataxia; (2) due to the development of malignant epidural spinal cord compression; or (3) due to the development of transient moderate dysphagia with a 3½ h duration that was probably related to the studied drug.
Hagen et al., 2011 [40] MSCP	Most AEs were described as mild (82%) or moderate (13%) in severity; all were well tolerated and had short durations (from 20 min to 1 h). Close to half of the patients described mild peri-oral tingling or numbness. Transient nausea was also reported by approximately one third of patients.	Four serious AEs were reported. Only one was related to the studied drug. The patient started taking the studied drug at a dose of 30 μg BID, and an hour after the second dose on day 1, she experienced hypertension and dizziness. Both events resolved the same day, and the patient was discharged.
Hagen et al., 2017 [41] Phase III MSCP	AEs were generally mild to moderate and transient. In the TTX group, all 77 patients experienced at least one AE that was considered drug-related. The most common were nausea, dizziness, oral numbness/tingling, and injection site irritation.	There were 12 serious AEs. Five AEs that occurred in three patients were probably related to TTX: ataxia (2), nystagmus (1), other neurotoxicity (1), and aspiration pneumonia (1). The aspiration pneumonia occurred in a patient who was at risk of aspiration.
Goldlust et al., 2021 [42] Phase II CINP	Across the TTX cohorts, 80.0 to 92.3% of the patients experienced at least one AE. The most frequent were oral paresthesia (34/100 TTX patients) and oral hypesthesia (28/100 TTX patients). Other common AEs were headache (19/100 TTX patients) and nausea (13/100 TTX patients).	Four patients had serious AEs, three of which were possibly related to treatment (hypertension, paresthesia, extremity pain, and a burning sensation). Two patients withdrew due to AEs: one patient with moderate vertigo and another with vertigo and an influenza-like illness, which were possibly related.

SCP: severe cancer pain; MSCP: moderate to severe cancer pain; CINP: chemotherapy-induced neuropathic pain; BID: twice daily; TID: three times a day.

## Data Availability

The full code used to perform the meta-analysis is available in the Supplementary Materials. Other data supporting the findings of this study are available from the corresponding author upon reasonable request.

## Supplementary Materials

The following supporting information can be downloaded at https://www.mdpi.com/article/10.3390/md21050316/s1, Data S1: Risk of Bias assessed with ROBINS-I; Data S2: Code used in R for the meta-analysis.
