# Supplementary material for: Efficacy and Security of Tetrodotoxin in the Treatment of Cancer-Related Pain: Systematic Review and Meta-Analysis
Source: Mar Drugs. 2023 May 21;21(5):316. doi: 10.3390/md21050316 (PMC10221257; doi:10.3390/md21050316)
Supplement: Supplementary file 1 [file marinedrugs-21-00316-s001.zip › marinedrugs-2385838-supplementary.pdf]

Supplementary Data 1: Risk of Bias assessed with ROBINS-I

Study	Domain 1	Domain 2	Domain 3	Domain 4	Domain 5	Domain 6	Domain 7	Overall
Hagen et al., 2007	Low	Moderate	Moderate	Low	Low	Moderate	Moderate	Moderate
Hagen et al., 2011	Low	Serious	Moderate	Low	Moderate	Moderate	Moderate	Serious

Table S1: Report of the consensus of the risk of bias with Risk Of Bias In Non-Randomized Studies of Interventions (ROBINS-I) tool evaluated by two authors. Domain 1: Bias due to confounding, Domain 2: Bias in selection of participants into the study, Domain 3: Bias in classification of interventions, Domain 4: Bias due to deviations from intended interventions, Domain 5: Bias due to missing data, Domain 6: Bias in measurement of outcomes, Domain 7: Bias in selection of the reported result.

Supplementary Data 2: Code used in R for the meta-analysis

META-ANALYSIS 1, EFFICACY:

```
 dat1 <- escalc(measure="OR", ai=dat.bcg$`TTX responders`, bi=dat.bcg$`TTX non-responders`,
ci=dat.bcg$`Placebo responders`, di=dat.bcg$`Placebo non-responders`, data=dat.bcg)
 dat1

            Author Year TTX.responders TTX.non.responders Placebo.responders Placebo.non.responders
yi     vi
1    Hagen et al., 2017           33              32              29                 55 0.6708 0.1142
2 Goldlust et al., 2021          15              11               8                 17  1.0639 0.3414
3    Hagen et al., 2008           16              25              12                 29 0.4361 0.2203

res1 <- rma(yi, vi, data=dat1)
Res1
confint(res1)

       estimate  ci.lb   ci.ub
tau^2    0.0000 0.0000  3.6863
tau      0.0000 0.0000  1.9200
I^2(%)   0.0000 0.0000 94.7028
H^2      1.0000 1.0000 18.8777

> res1
Random-Effects Model (k = 3; tau^2 estimator: REML)

tau^2 (estimated amount of total heterogeneity): 0 (SE = 0.1980)
tau (square root of estimated tau^2 value):      0
I^2 (total heterogeneity / total variability):   0.00%
H^2 (total variability / sampling variability):  1.00
```

```
Test for Heterogeneity:
Q(df = 2) = 0.7023, p-val = 0.7039

Model Results:

estimate      se    zval    pval   ci.lb   ci.ub
  0.6761  0.2483  2.7233  0.0065  0.1895  1.1627  **

---
Signif. codes:  0 ‘***’ 0.001 ‘**’ 0.01 ‘*’ 0.05 ‘.’ 0.1 ‘ ’ 1
```

```
forest(res1, xlim=c(-1.8,4), annotate=TRUE, showweights=TRUE, col = "green", border = "black", cex=1.6, order="obs", header="Author(s), Year", slab=paste(dat.bcg$Author, dat.bcg$Year, sep=", "))
```

META-ANALYSIS 2, >1 AE RELATED TO THE DRUG:

Author	Year	TTX AE	TTX NON-AE	PLACEBO AE	PLACEBO NON-AE
Hagen et al.,	2017	77	0	77	11
Goldlust et al.,	2021	9	17	3	22
Hagen et al.,	2008	34	7	28	12

```
datAE <- escalc(measure="OR", ai=X_1_AE_related_to_the_drug$`TTX AE`, bi=X_1_AE_related_to_the_drug$`TTX NON-AE`, ci=X_1_AE_related_to_the_drug$`PLACEBO AE`, di=X_1_AE_related_to_the_drug$`PLACEBO NON-AE`, data=X_1_AE_related_to_the_drug)
> dat1

          Author Year TTX.responders TTX.non.responders Placebo.responders Placebo.non.responders
yi     vi
1    Hagen et al., 2017            33               32               29                   55 0.6708 0.1142
2 Goldlust et al., 2021            15               11                8                   17 1.0639 0.3414
3    Hagen et al., 2008            16               25               12                   29 0.4361 0.2203

> resAE=rma(datAE$yi, datAE$vi, data=datAE)
> resAE
```

Random-Effects Model (k = 3; tau^2 estimator: REML)

tau^2 (estimated amount of total heterogeneity): 0.0000 (SE = 0.5672)
tau (square root of estimated tau^2 value):   0.0026
I^2 (total heterogeneity / total variability):   0.00%
H^2 (total variability / sampling variability): 1.00

Test for Heterogeneity:
Q(df = 2) = 2.5377, p-val = 0.2812

forest(resAE, annotate=TRUE, showweights=TRUE, col = "red", border = "black", cex=1.6, order="obs", header="Author(s), Year", slab=paste(X_1_AE_related_to_the_drug$Author, X_1_AE_related_to_the_drug$Year, sep=", "))

META-ANALYSIS 3, SERIOUS ADVERSE EVENTS RELATED TO THE DRUG

```
 SAE_related_to_the_drug <- read_excel("C:/Users/ASUS/Downloads/SAE related to the drug.xlsx")
> View(SAE_related_to_the_drug)
> datSAE <- escalc(measure="OR", ai=SAE_related_to_the_drug$`TTX SAE`,
bi=SAE_related_to_the_drug$`TTX NON-SAE`, ci=SAE_related_to_the_drug$`PLACEBO SAE`,
di=SAE_related_to_the_drug$`PLACEBO NON-SAE`, data=SAE_related_to_the_drug)
> datSAE
```

	Author	Year	TTX.SAE	TTX.NON.SAE	PLACEBO.SAE	PLACEBO.NON.SAE	yi	vi
1	Hagen et al.,	2017	6	71	3	85	0.8731	0.5258
2	Goldlust et al.,	2021	1	25	1	24	-0.0408	2.0817
3	Hagen et al.,	2008	1	40	0	41	1.1230	2.7155

```
resSAE=rma(datAE$yi, datAE$vi, data=datAE)
> resSAE
```

Random-Effects Model (k = 3; tau^2 estimator: REML)

tau^2 (estimated amount of total heterogeneity): 0 (SE = 1.4420)
tau (square root of estimated tau^2 value):   0
I^2 (total heterogeneity / total variability):   0.00%
H^2 (total variability / sampling variability): 1.00

Test for Heterogeneity:
Q(df = 2) = 0.3805, p-val = 0.8268

Model Results:

estimate	se	zval	pval	ci.lb	ci.ub
0.7469	0.6030	1.2387	0.2154	-0.4349	1.9288

Signif. codes: 0 '***' 0.001 '**' 0.01 '*' 0.05 '.' 0.1 ' ' 1
